# Predicting Protein-Protein Interactions from Primary Protein Sequences Using a Novel Multi-Scale Local Feature Representation Scheme and the Random Forest

**DOI:** 10.1371/journal.pone.0125811

**Published:** 2015-05-06

**Authors:** Zhu-Hong You, Keith C. C. Chan, Pengwei Hu

**Affiliations:** 1 Department of Computing, The Hong Kong Polytechnic University, Hong Kong, China; 2 School of Electronics and Information Engineering, Tongji University, Shanghai, China; London, UNITED KINGDOM

## Abstract

The study of protein-protein interactions (PPIs) can be very important for the understanding of biological cellular functions. However, detecting PPIs in the laboratories are both time-consuming and expensive. For this reason, there has been much recent effort to develop techniques for computational prediction of PPIs as this can complement laboratory procedures and provide an inexpensive way of predicting the most likely set of interactions at the entire proteome scale. Although much progress has already been achieved in this direction, the problem is still far from being solved. More effective approaches are still required to overcome the limitations of the current ones. In this study, a novel Multi-scale Local Descriptor (MLD) feature representation scheme is proposed to extract features from a protein sequence. This scheme can capture multi-scale local information by varying the length of protein-sequence segments. Based on the MLD, an ensemble learning method, the Random Forest (RF) method, is used as classifier. The MLD feature representation scheme facilitates the mining of interaction information from multi-scale continuous amino acid segments, making it easier to capture multiple overlapping continuous binding patterns within a protein sequence. When the proposed method is tested with the PPI data of *Saccharomyces cerevisiae*, it achieves a prediction accuracy of 94.72% with 94.34% sensitivity at the precision of 98.91%. Extensive experiments are performed to compare our method with existing sequence-based method. Experimental results show that the performance of our predictor is better than several other state-of-the-art predictors also with the *H*. *pylori* dataset. The reason why such good results are achieved can largely be credited to the learning capabilities of the RF model and the novel MLD feature representation scheme. The experiment results show that the proposed approach can be very promising for predicting PPIs and can be a useful tool for future proteomic studies.

## Introduction

Protein-protein interactions (PPIs) play a key role in various biological processes and functions in living cells, including metabolic cycles, DNA transcription and replication, and signalling cascades [1–3]. Thus, correctly identifying and characterizing protein interactions are critical for understanding the molecular mechanisms inside the cell [3]. In the past decades, many innovative experimental techniques for detecting PPI have been developed [3–5]. Due to the progress in large-scale experimental technologies such as yeast two-hybrid (Y2H) screens [4,6], tandem affinity purification (TAP) [3], mass spectrometric protein complex identification (MS-PCI) [5] and other high-throughput biological techniques for PPI detection, an immense amount of PPI data for different species has been accumulated [3–7].

However, the experimental methods are costly and time consuming, therefore current PPI pairs obtained with experimental methods covers only a small fraction of the complete PPI networks [8,9]. In addition, large-scale experimental methods usually suffer from high rates of both false positive and false negative predictions [10]. Hence, it is of great practical significance to develop the reliable computational methods to facilitate identification of PPI [11,12].

A number of computational techniques have been proposed to provide either complementary information or supporting evidence to experimental methods [13–16]. Existing approaches typically use binary classification frameworks that differ in the features used to represent protein pairs. Different protein attributes or feature sources, such as protein structure information[17,18], protein domains, gene neighbourhood, phylogenetic profiles, gene expression, and literature mining knowledge are employed to infer protein interactions[8,19–22]. There are also methods that combine interaction information from several different data sources[23]. However, these methods cannot be implemented if such pre-knowledge about the proteins is not available[24,25].

Recently, a couple of methods which derive information directly from amino acid sequence are of particular interest [12,20,26–30]. Many researchers have engaged in the development of sequences-based method for discovering new PPI[31–33], and the experimental results showed that the information of amino acid sequences alone is sufficient to predict PPI[12,20,34]. Among them, one of the excellent works is a SVM-based method developed by Shen et al [12]. In the study, the 20 amino acids are clustered into seven classes according to their dipoles and volumes of the side chains, and then the conjoint triad method abstracts the features of protein pairs based on the classification of amino acids. When applied to predict human PPI, this method yields a high prediction accuracy of 83.9%. Because the conjoint triad method cannot takes neighbouring effect into account and the interactions usually occur in the discontinuous amino acids segments in the sequence, on the other work Guo et al. developed a method based on SVM and auto covariance to extract the interactions information in the discontinuous amino acids segments in the sequence [35]. Their method yielded a prediction accuracy of 86.55%, when applied to predicting *saccharomyces cerevisiae* PPI. In our previous works, we also obtained good prediction performance by using autocorrelation descriptors and correlation coefficient, respectively [26,36].

In this study, a novel feature representation method for prediction of PPI is proposed. We hypothesize that the continuous amino acids segments with different segment lengths play an important role in determining the interactions between proteins. In other words, the proposed protein representation method gives adequate consideration to mine the interaction information from multi-scale continuous amino acid segments at the same time, thus it can sufficiently capture multiple overlapping continuous binding patterns within a protein sequence.

To sum up, in this paper we propose a sequence-based approach for the prediction of protein-protein interactions using random forest (RF) model combined with a novel multi-scale local descriptor (MLD) protein feature representation. To evaluate the performance, the proposed method is applied to *Saccharomyces cerevisiae* PPI dataset. The experiment results show that our method achieved 94.72% prediction accuracy with 94.34% sensitivity at the precision of 98.91%. The prediction model is also assessed using the independent dataset of the *Helicobacter pylori* PPI and yielded 88.30% prediction accuracy, which further demonstrates the effectiveness of our method.

## Results

In this section, we first discuss the biological datasets and evaluation strategies used in performance comparisons. Next we present results for comparing the proposed method to state-of-the-art sequence-based method for predicting protein interaction pairs in yeast.

### Protein sequence and protein interaction dataset

To evaluate the performance of the proposed approach, there are a total of 8 different PPI datasets are used in our experiments, two of which are *S*.*cerevisiae*, two are *H*. *pylori*, one is *C*.*elegans*, one is *E*.*coli*, one is *H*.*sapiens*, and one is *M*.*musculus*.

The PPI dataset which were derived by Guo *et al*.[35], are used to build the first prediction model. The dataset was downloaded from *S*.*cerevisiae* core subset of database of interacting proteins (DIP) [37]. After the protein pairs that contain a protein with fewer than 50 residues or have more than 40 percent sequence identity were removed, the remaining 5594 protein pairs formed the golden standard positive dataset (GSP). The construction of a negative PPI dataset is very important for training and evaluating prediction model. However, it is difficult to generate such a dataset because we have limited information about proteins that are really non-interactive. Here, the negative dataset is generated by firstly selecting non-interacting pairs uniformly at random from the set of all proteins pairs that are not known to interact. Then the protein pairs with same subcellular localization information are excluded. Finally, the golden standard negative dataset (GSN) consisted of 5594 protein pairs whose subcellular localization is different. By combining the above GSP and GSN datasets, the complete dataset contains of 11188 protein pairs, where half are from the positive dataset and half from the negative dataset. Note that here we have used exactly the same PPI dataset as used in Guo et al [35]. The names of protein pairs and their sequences of the dataset are given in online supplementary material at https://sites.google.com/site/zhuhongyou/data-sharing.

However, some researchers argue that restricting negative examples to protein pairs localized in different cellular compartments is not appropriate for evaluating classifier accuracy [38,39]. The use of such negative dataset for building a model can result primarily in predictions of protein co-localization [40]. The fact that interacting protein pairs have to be in the same place does not mean that all proteins in the same compartment will be interacting with each other. Therefore, we constructed the second PPI dataset by using positive samples from first PPI dataset, and following simpler selection scheme—choosing negative examples uniformly at random—to construct the negative dataset. The second PPI dataset also consists of 11188 protein pairs, where half are from the positive dataset and half from the negative dataset.

The third PPI dataset is composed of 2916 *Helicobacter pylori* protein pairs (1458 interacting pair and 1458 non-interacting pairs) as described by Martin et al [41]. Other five species-specific PPI dataset including *C*.*elegans*, *E*.*coli*, *H*.*sapiens*, *M*.*musculus*, and *H*.*pylori* are employed in our experiment to verify the effectiveness of the proposed method.

### Evaluation measures

To measure the performance of the proposed method, we adopt five-fold cross validation and a couple of validation measures in this study. These criteria are as follows: (1) the overall prediction accuracy (ACC) is the percentage of correctly identified interacting and non-interacting protein pairs and given by:

$$ACC=\frac{TP+TN}{TP+FP+TN+FN}$$ (1)

(2) the sensitivity (SN) is the percentage of correctly identified interacting protein pairs and given by:

$$SN=\frac{TP}{TP+FN}$$ (2)

(3) the specificity (Spec) is the percentage of correctly identified non-interacting protein pairs and given by:

$$Spec=\frac{TN}{TN+FP}$$ (3)

(4) the positive predictive value (PPV) is the positive prediction value and given by:

$$PPV=\frac{TP}{TP+FP}$$ (4)

(5) the negative predictive value (NPV) is the negative prediction value and given by:

$$NPV=\frac{TN}{TN+FN}$$ (5)

(6) the F_score_ is a weighted average of the PPV and sensitivity, where an F_score_ reaches its best value at 1 and worst score at 0; The definitions is given as follows:

$$F_{score}=2\times \frac{SN\times PPV}{SN+PPV}$$ (6)

(7) the Matthew’s correlation coefficient (MCC) is more stringent measure of prediction accuracy accounts for both under and over-predictions. Its definitions is given by:

$$MCC=\frac{TP\times TN-FP\times FN}{\sqrt{\left(TP+FN)\times (TN+FP)\times (TP+FP)\times (TN+FN\right)}}$$ (7)

where true positive (TP) is the number of true PPIs that are predicted correctly; false negative (FN) is the number of true PPIs that are predicted to be non-interacting pairs; false positive (FP) is the number of true non-interacting pairs that are predicted to be PPIs, and true negative (TN) is the number of true non-interacting pairs that are predicted correctly.

### Experimental setting

In this paper, the proposed sequence-based PPI predictor is implemented using MATLAB platform. All the simulations are carried out on a computer with 3.1 GHz 2-core CPU, 6 GB memory and Windows operating system. In order to achieve good experimental results, the corresponding parameters for random forest are firstly optimized. For RF model the parameters to be ascertained are the number of feature subset *M*, and the ensemble size *N*. The average prediction results for six testing datasets are listed in Table 1 by setting *M* to 5, 10, 15, 20, 25 and 30, respectively. It can be found that the performance under different conditions varies slightly, and none of the parameters take obvious advantage over the other ones. So there is no consistent relationship between the classification accuracy and feature subset *M*. In this study, the value of *M* is set to 10 in all experiments, which requires the relatively less computational cost.

**Table 1:** The prediction performance for six testing datasets with various number of feature subsets *M*, where the tree size *N* is set to 60.

**M**	ACC*(%)*	SN*(%)*	Spec	PPV*(%)*	NPV*(%)*	F1*(%)*	MCC*(%)*
5	94.72±0.35	94.45±0.55	95.72±1.51	98.80±0.43	82.17±1.27	96.58±0.25	85.89±0.70
10	94.63±0.37	94.42±0.54	95.40±1.29	98.71±0.38	82.04±1.32	96.52±0.26	85.65±0.76
15	94.62±0.21	94.44±0.53	95.28±1.58	98.69±0.45	82.06±1.19	96.51±0.15	85.60±0.41
20	94.60±0.36	94.35±0.54	95.56±1.68	98.76±0.48	81.88±1.23	96.50±0.25	85.61±0.76
25	94.69±0.25	94.44±0.44	95.66±1.34	98.79±0.39	82.11±0.99	96.56±0.18	85.82±0.47
30	94.63±0.37	94.42±0.54	95.39±1.43	98.71±0.41	82.04±1.25	96.52±0.26	85.65±0.78

**Fig 1:**
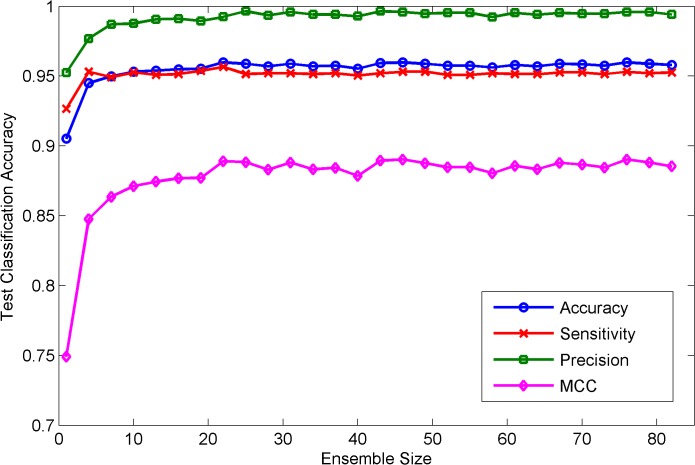
The prediction performance for classification accuracy, sensitivity, precision and MCC of different tree size, where the number of feature subsets M is set to 10 and the unpruned decision tree is employed as the base classifier.

The average results with different ensemble sizes are shown in Fig 1. It can be found from Fig 1 that RF predictor performs well when only a few of base classifiers are employed. All the evaluation measures including average prediction accuracy, sensitivity, specificity, PPV, NPV and MCC keep improving with ensemble size increase. However, the improvement becomes negligible when the ensemble size is larger than 10. From the above analyses, we can conclude that RF model is not sensitive to the choice of parameters. So for the *H*.*pylori* dataset, the parameters of RF model do not need to be optimized again, assuming that they are set the same values as those adopted on the *S*.*cerevisiae* dataset.

### Prediction performance of proposed model

We evaluated the performance of the proposed model using the first PPIs dataset as investigated in Guo *et al*. [35]. To guarantee that the experimental results are valid and can be generalized for making predictions regarding new data, the dataset is randomly partitioned into training and independent testing sets via a five-fold cross validation. Each of the five subsets acts as an independent holdout testing dataset for the model trained with the rest of four subsets. Thus five models are generated for the five sets of data. The advantages of cross validation are that the impact of data dependency is minimized and the reliability of the results can be improved.

The prediction performance of RF predictor with MLD representation of protein sequence across five runs is shown in Table 2. It can be observed from Table 2 that high prediction accuracy of 94.72% is obtained for the proposed model. To better investigate the prediction ability of our model, we also calculated the values of Sensitivity, Positive Predictive Value, and MCC. From Table 2, we can see that our model gives good prediction performance with an average sensitivity value of 94.34%, PPV value of 98.91%, accuracy value of 94.72%, and MCC value of 85.99%. Further, it can also be seen in the Table 2 that the standard deviation of sensitivity, PPV, accuracy, and MCC are as low as 0.0049, 0.0033, 0.0043, and 0.0089, respectively.

**Table 2:** Comparison of the prediction performance by the proposed method and some state-of-the-art works on the yeast dataset.

*Model*	*Features*	*Classifier*	*SN(%)*	*PPV(%)*	*ACC(%)*	*MCC(%)*
Our method *(1st dataset)*	MLD	RF	**94.34±0.49**	**98.91±0.33**	**94.72±0.43**	**85.99±0.89**
Our method *(2nd dataset)*	MLD	RF	92.67±0.79	99.51±0.23	93.83±0.61	84.05±1.47
Guo’s work *(1st dataset)*	ACC	SVM	89.93±3.68	88.87±6.16	89.33±2.67	N/A
	AC	SVM	87.30±4.68	87.82±4.33	87.36±1.38	N/A
Zhou’s work *(1st dataset)*	LD	SVM	87.37±0.22	89.50±0.60	88.56±0.33	77.15±0.68
Yang’s work *(1st dataset)*	LD (Cod1)	KNN	75.81±1.20	74.75±1.23	75.08±1.13	N/A
	LD (Cod2)	KNN	76.77±0.69	82.17±1.35	80.04±1.06	N/A
	LD (Cod3)	KNN	78.14±0.90	81.86±0.99	80.41±0.47	N/A
	LD (Cod4)	KNN	81.03±1.74	90.24±1.34	86.15±1.17	N/A

Here, N/A means not available.

For the first PPI dataset, we define negative examples exploiting the fact that proteins from different cellular locations are unlikely to interact [42]. However, it was shown that this approach, when used to train PPI prediction methods, leads to a bias in the estimation of prediction accuracy, since the additional constraints related to localization make the prediction task easier [38]. Another typical choice is to select non-interacting pairs uniformly at random from the set of all proteins pairs that are not known to interact. Therefore, in our experiments we also use the second PPI dataset to verify the effectiveness of the proposed method. Table 2 illustrates the comparison of the prediction performance using two kinds of negative sample selection methods on the *yeast* dataset. As shown in the table, the performance of first PPI dataset (selecting negative examples using cellular localization information) is slightly better than that of the second PPI dataset (randomly selected negative examples without cellular localization information). We can explain the higher accuracy for the first PPI dataset by the fact that the constraint on localization restricts the negative examples to a sub-space of feature space, making the learning problem easier than when there is no constraint.

We further compared our method with Guo et al.[35], Zhou et al.[43] and Yang et al.[44], where the SVM, SVM and KNN is performed with the Auto Covariance (or Auto Cross Covariance), Local Descriptor, and Local Descriptor with four kinds of coding scheme as the input feature vectors, respectively. From Table 2, we can see that the performance of all of these methods with different machine learning model and sequence-based feature representation are lower than ours, which indicates the advantages of our method. To sum up, we can readily conclude that the proposed approach generally outperforms the previous model with higher discrimination power for predicting PPIs based the information of protein sequences. Therefore, we can see clearly that our model is a much more appropriate method for predicting new protein interactions compared with the other methods. Consequently, it makes us be more convinced that the proposed method can be very helpful in assisting the biologist to assist in the design and validation of experimental studies and for the prediction of interaction partners.

### Comparing the prediction performance of ensemble classifier with single classifier methods

**Fig 2:**
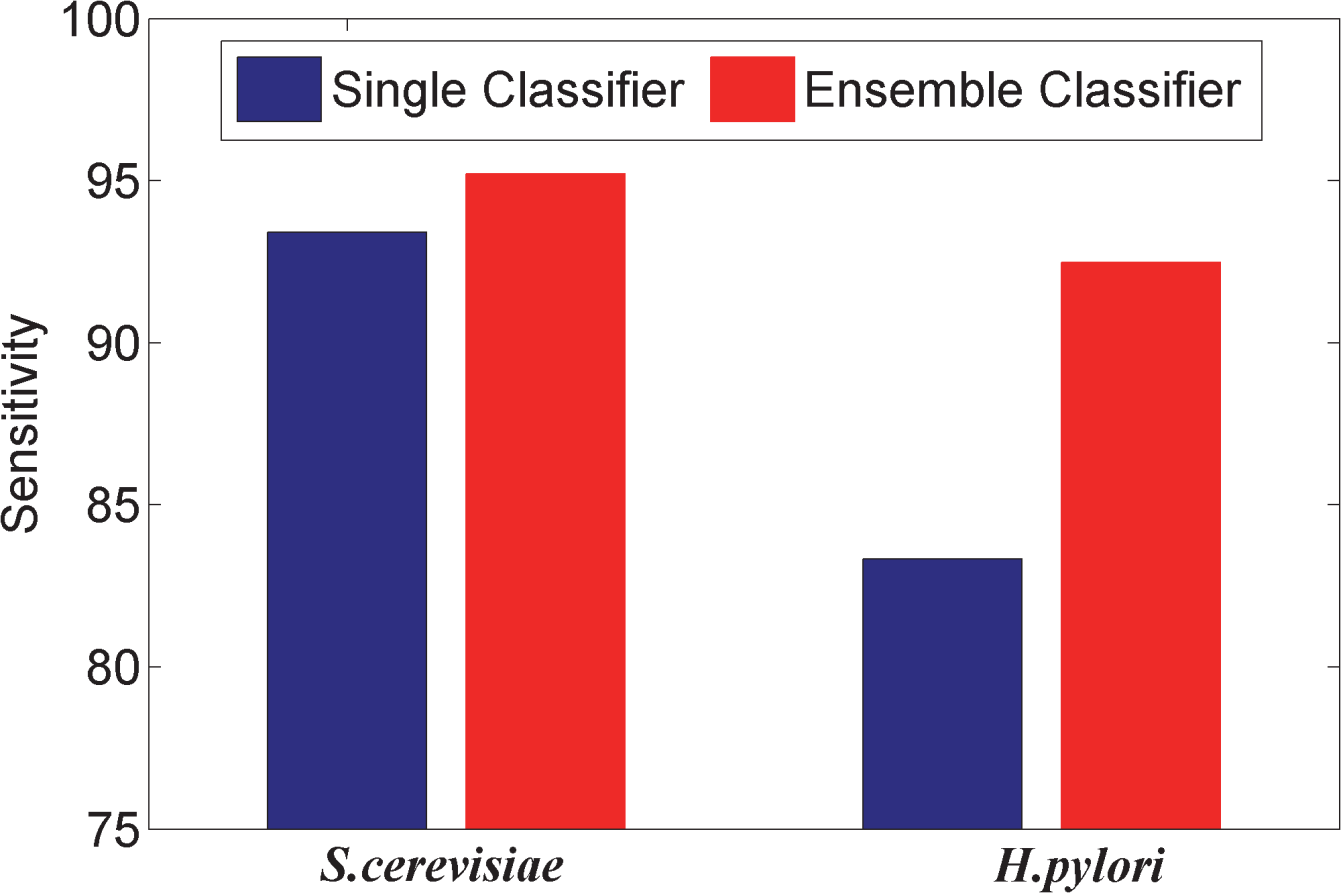
Comparison for the Sensitivity value of the ensemble classifier versus single classifiers on the dataset of *S*.*cerevisiae* and *H*.*pylori*.

**Fig 3:**
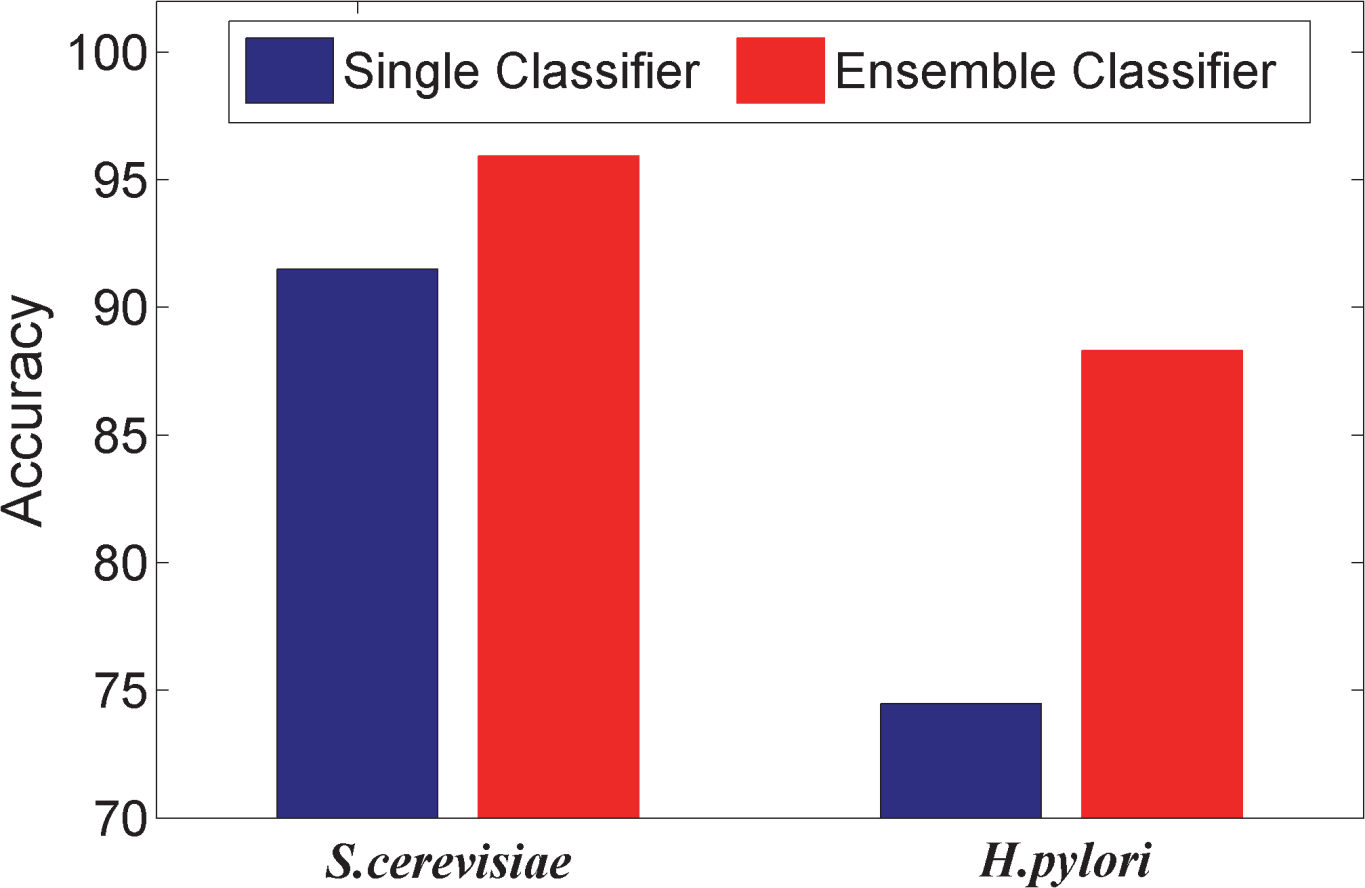
Comparison for the Accuracy value of the ensemble classifier versus single classifiers on the dataset of *S*.*cerevisiae* and *H*.*pylori*.

**Fig 4:**
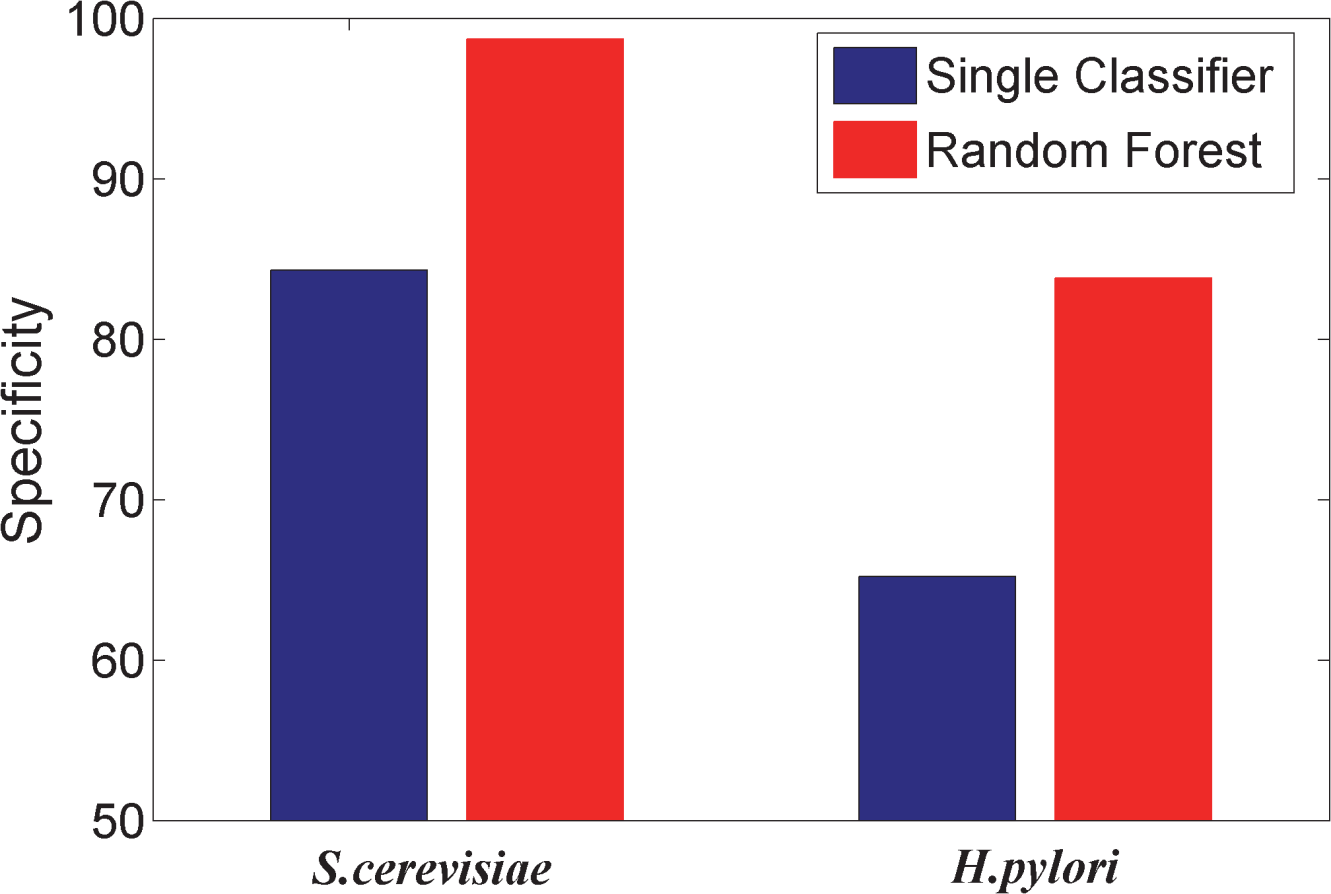
Comparison for the Specificity value of the ensemble classifier versus single classifiers on the dataset of *S*.*cerevisiae* and *H*.*pylori*.

**Fig 5:**
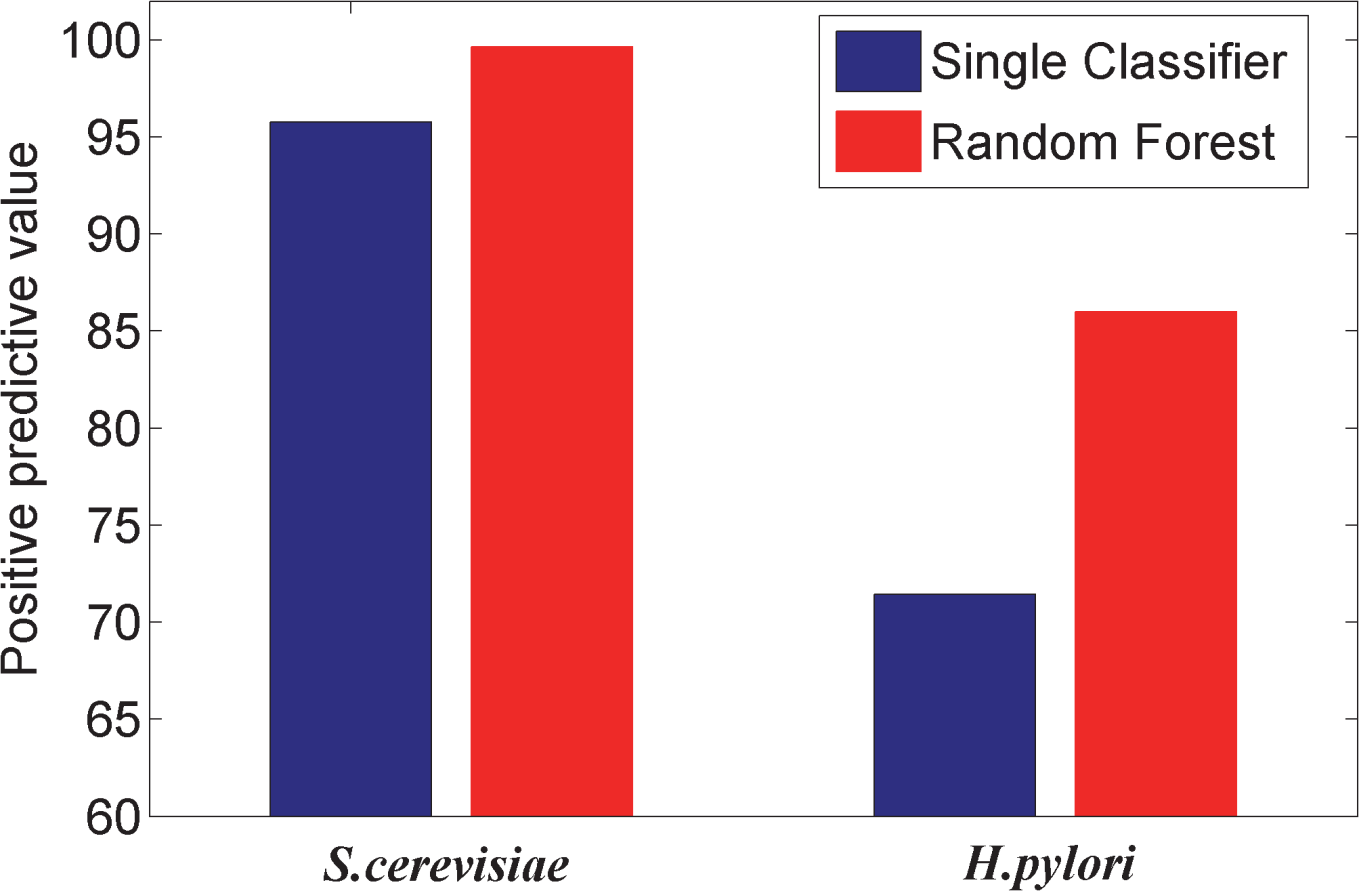
Comparison for the Predictive PositiveV value of the ensemble classifier versus single classifiers on the dataset of *S*.*cerevisiae* and *H*.*pylori*.

**Fig 6:**
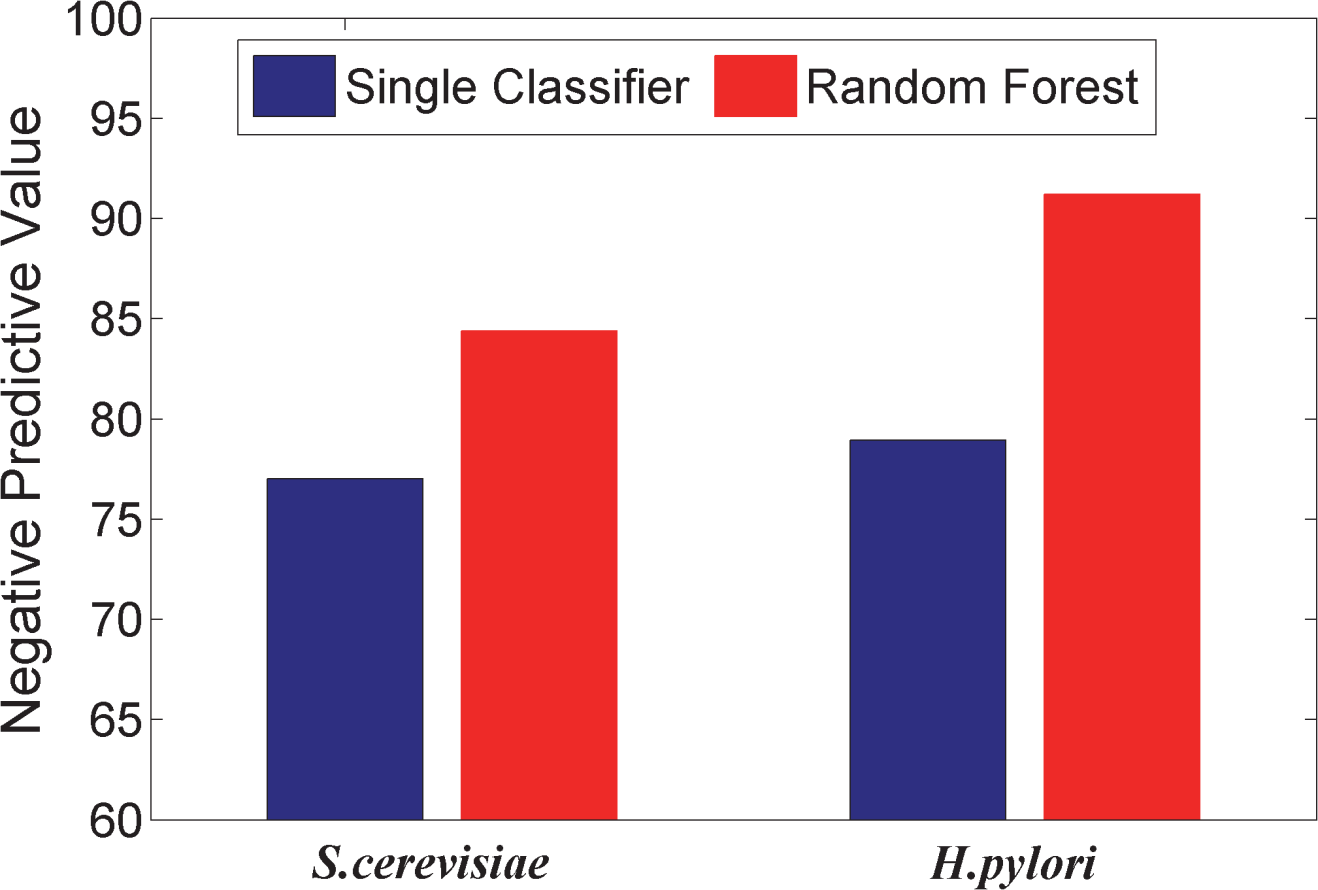
Comparison for the Negative Positive Value of the ensemble classifier versus single classifiers on the dataset of *S*.*cerevisiae* and *H*.*pylori*.

**Fig 7:**
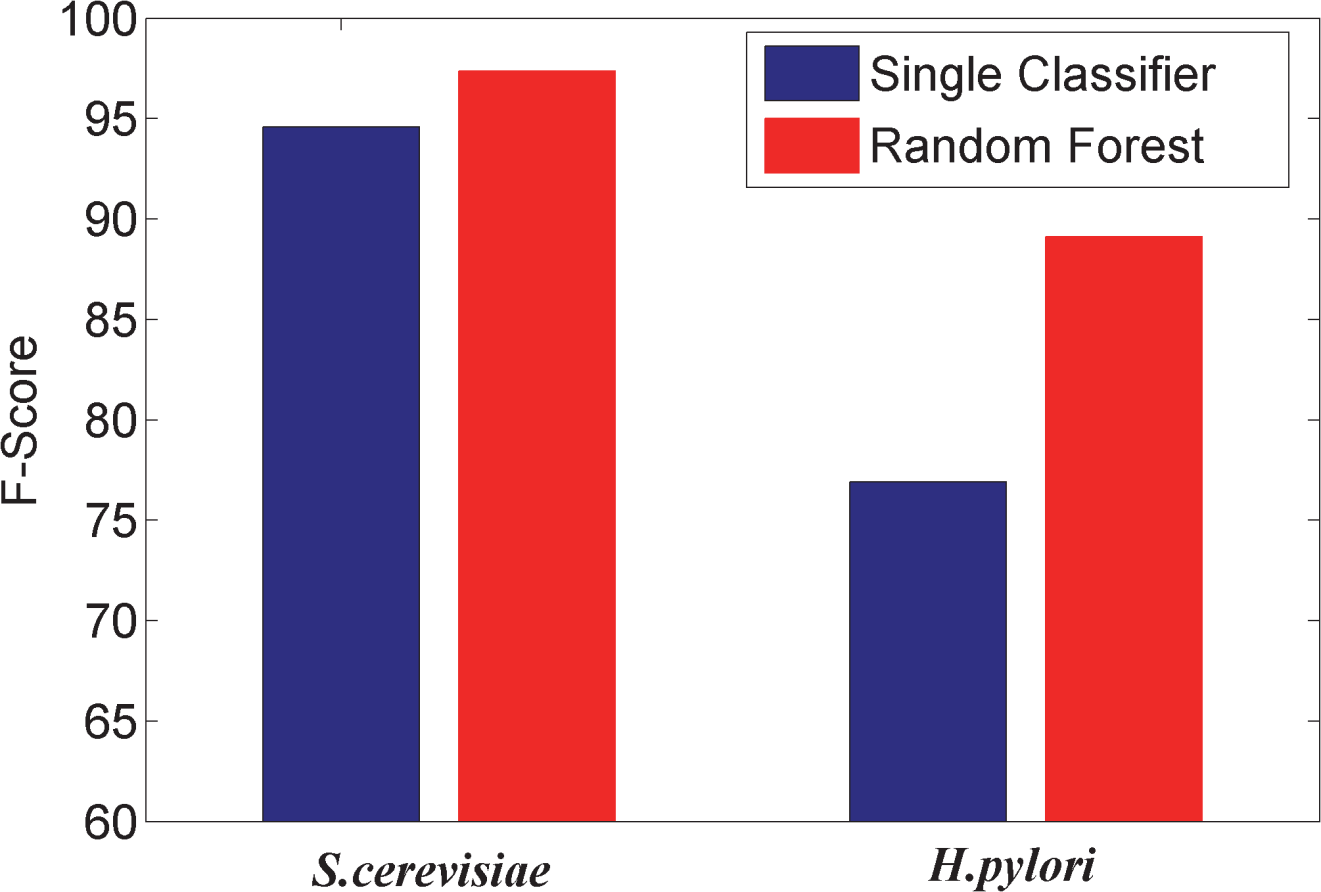
Comparison for the F-Score value of the ensemble classifier versus single classifiers on the dataset of *S*.*cerevisiae* and *H*.*pylori*.

**Fig 8:**
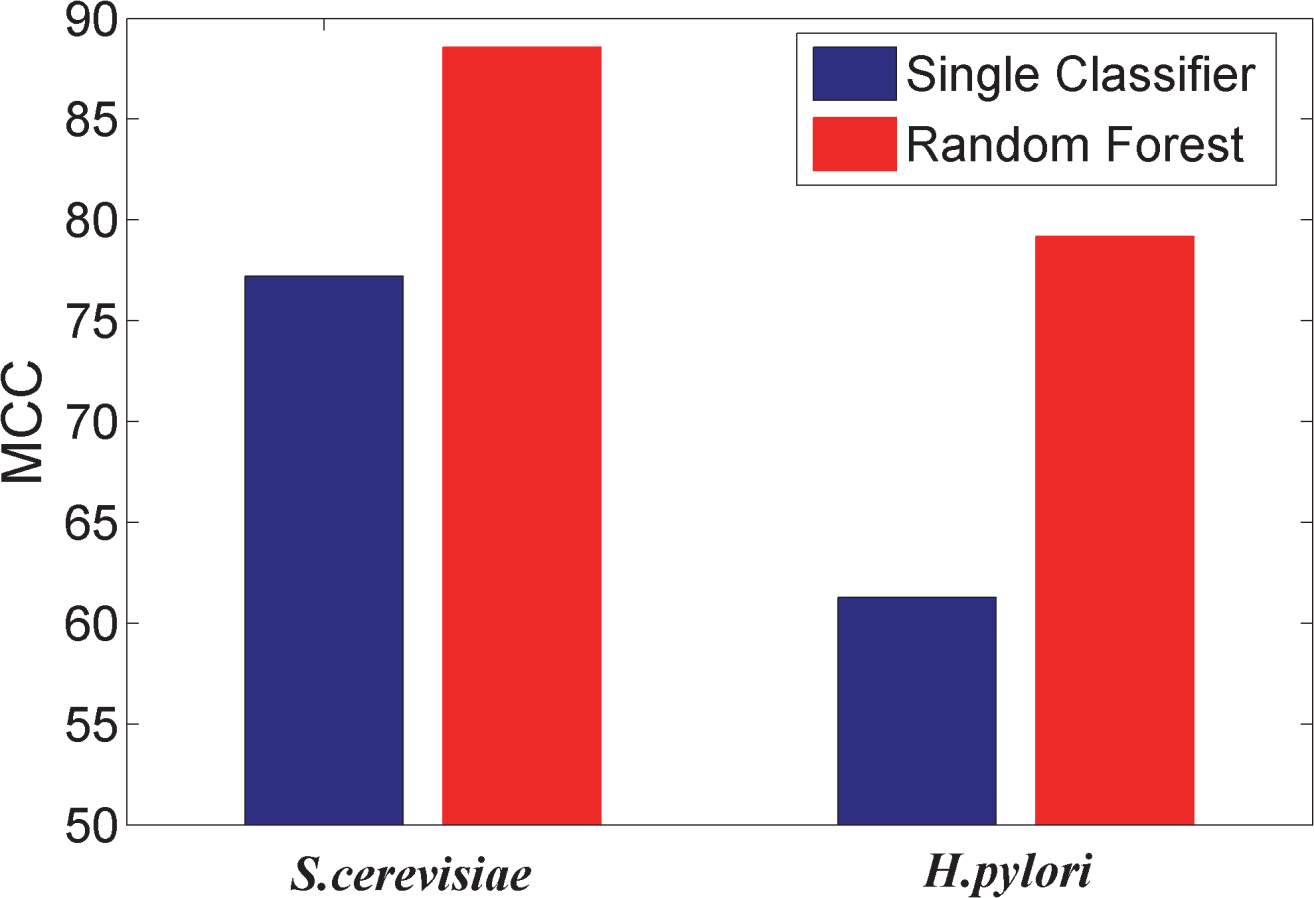
Comparison for the Matthews Correlation Coefficient value of the ensemble classifier versus single classifiers on the dataset of *S*.*cerevisiae* and *H*.*pylori*.

We here investigated whether or not the ensemble of classifiers can significantly improve the performance of PPI prediction compared against the individual classifier in the ensemble. Figs 2–8 plots the sensitivity, accuracy, specificity, PPV, NPV, F-Score, and MCC values for the component classifiers decision tree and the ensemble classifier random forest. The results in Figs 2–8 clearly demonstrate that the ensemble classifier dominates the component classifiers. The PPV value obtained by the ensemble classifier is nearly 4.3% higher than the component classifier on the *S*.*cerevisiae* dataset. In addition, the sensitivity is improved from 92.66% to 95.15% while the accuracy is improved from 90.52% to 95.80%. Further, on the *H*.*pylori* dataset, it can also be seen from the Figs 2–8 that the ensemble classifier dominates the single classifier. We concluded that the ensemble classifier is much more accurate than the single classifier that makes them up.

### Comparing the prediction performance between our method and other existing methods

In order to highlight the advantage of our model, it is also tested by *Helicobacter pylori* dataset. The *H*. *pylori* dataset is composed of 2,916 protein pairs (1,458 interacting pair and 1,458 non-interacting pairs) as described by Martin *et al* [45]. This dataset gives a comparison of proposed method with other previous works including phylogenetic bootstrap[46], signature products[45], HKNN[47], ensemble of HKNN[48] and boosting. The methods of phylogenetic bootstrap, signature products and HKNN are based on individual classifier system to infer PPI, while the methods of HKNN and boosting belong to ensemble-based classifiers. The average prediction performances of ten-fold cross-validation over six different methods are shown in Table 3. From Table 3, we can see that the average prediction performance, i.e. sensitivity, PPV, accuracy and MCC achieved by proposed predictor, are 92.47%, 85.99%, 88.30% and 79.19%, respectively. It demonstrates that our method outperforms all other individual classifier-based methods and the ensemble classifier systems (i.e. ensemble of HKNN and Boosting). All these results show that the proposed method not only achieves accurate performance, but also substantially improves positive predictive value in the prediction of PPI.

**Table 3:** Performance comparison of different methods on the *H*.*pylori* dataset.

Methods	Sensitivity	PPV	Accuracy	MCC
**Phylogenetic bootstrap**	69.80**%**	80.20**%**	75.80**%**	N/A
**HKNN**	86.00**%**	84.00**%**	84.00**%**	N/A
**Signature products**	79.90**%**	85.70**%**	83.40**%**	N/A
**Ensemble of HKNN**	86.70**%**	85.00**%**	86.60**%**	N/A
**Boosting**	80.37**%**	81.69**%**	79.52**%**	70.64**%**
**Proposed method**	**92.47%**	**85.99%**	**88.30%**	**79.19%**

Here, N/A means not available.

### Prediction performance on the independent *Yeast* datasets

Since the proposed method achieved a high performance on *S*.*cerevisiae* and *H*.*pylori* datasets, we switch to evaluate the prediction performance of our approach on five independent testing datasets, which means using of experimentally identified interactions in one organism to predict the interactions in other organisms assuming that homolog proteins preserve their ability to interact (5,6). The basis of this hypothesis is to assume that homologs have similar functional behaviour; therefore, they preserve the same PPI [36]. Five independent datasets are employed to validate the quality of predicting PPIs which share low identity with the training dataset. Specifically, we first trained the prediction model on the entire *yeast* dataset. Then we performed blind test on five PPI datasets which are independent of the training dataset. The performance of our method for predicting five independent datasets is summarized in Table 4. As shown in the table, the proposed method gives good prediction performance with accuracy of 87.71%, 89.30%, 94.19%, 91.69%, and 90.99% on *C*.*elegans*, *E*.*coli*, *H*.*sapiens*, *M*.*musculus*, and *H*. *pylori*, respectively. Further, it can also be seen in the Table 4 that the sensitivity in *C*.*elegans*, *E*.*coli*, *H*.*sapiens*, *M*.*musculus*, *and H*. *pylori* are 87.71%, 89.30%, 94.19%, 91.69%, 90.99%, respectively. The F-Scores for these organisms are 93.46%, 94.35%, 97.01%, 95.67%, 95.28%, respectively. It demonstrates that the RF prediction model with MLD representation can achieve high prediction performance towards cross-species datasets. It should be noticed that the PPI dataset of *S*.*cerevisiae* is employed to construct the prediction model, so the trained model represented the characteristics of *S*.*cerevisiae* PPI. Meanwhile, our model can also represent the features of *C*.*elegans*, *E*.*coli*, *H*.*sapiens*, *M*.*musculus*, and *H*. *pylori*, which illustrated the good generalization ability of the proposed model on these organisms.

**Table 4:** Prediction performance on five species based on our model.

Species	# Test pairs	ACC (%)	SN (%)	PPV (%)	NPV (%)	F1
*C*.*elegans*	4013	87.71%	87.71%	100%	0	93.46%
*E*.*coli*	6954	89.30%	89.30%	100%	0	94.35%
*H*.*sapiens*	1412	94.19%	94.19%	100%	0	97.01%
*M*.*musculus*	313	91.69%	91.69%	100%	0	95.67%
*H*. *pylori*	1420	90.99%	90.99%	100%	0	95.28%

This finding indicates that the proposed model can be successfully applied to other species for which experimental PPI data is not available. It should be noticed that it is reasonable that PPIs generated in one species can be used to predict interactions in other species. The large numbers of PPIs in one organism might have “coevolved” with other organisms so that their corresponding orthologues interact as well [39]. We emphasized that this notion of conserved interactions, or “interologs”, is also supported by the observation that many interactions in signal transduction pathways or molecular machines are conserved between different species [49].

## Conclusions

In this paper, we develop an efficient technique for predicting protein interactions from protein sequences by combining a novel Multi-scale Local Descriptor (MLD) feature representation with RF model. The MLD representation takes into account the factors that PPI usually occurs in continuous segments with varying lengths in the protein sequence. In our study, protein sequences are characterized by a number of regions using MLD representation, which is capable of capturing multiple overlapping continuous binding patterns within a protein sequence. Experimental results demonstrated that the proposed method performed significantly well in distinguishing interacting and non-interacting protein pairs. Achieved results demonstrate that the proposed approach outperforms all other previous methods on a couple of PPI datasets and can be a useful supplementary tool to traditional experimental method.

## Methods

In this section, we describe the proposed MLD-RF approach for predicting protein interactions from protein sequences. Our method to predict the PPI depends on two steps: (1) Represent protein sequences as a vector by using the proposed MLD feature representation; (2) RF predictor is used to perform protein interactions prediction tasks.

### Feature Vector Extraction

In algorithm development, feature extraction is one of the most important components that significantly affect the performance of computational model. To successfully use the machine learning methods to predict PPI from protein sequences, one of the most important computational challenges is how to effectively represent a protein sequence by a fixed length feature vector in which the important information content of proteins is fully encoded. Although researchers have proposed various sequence-based methods to predict new PPI, one flaw of them is that the interactions information cannot be drawn from multi-scale continuous amino acids segments with different segment lengths at the same time. To overcome this shortcoming, in this study we propose a novel MLD sequence representation approach to transform the protein sequences into feature vectors by using a binary coding scheme. A multi-scale decomposition technique is used to divide protein sequence into multiple sequence segments of varying length to describe overlapping local regions. Here, the continuous sequence segments are composed of residues which are local in the polypeptide sequence.

**Fig 9:**
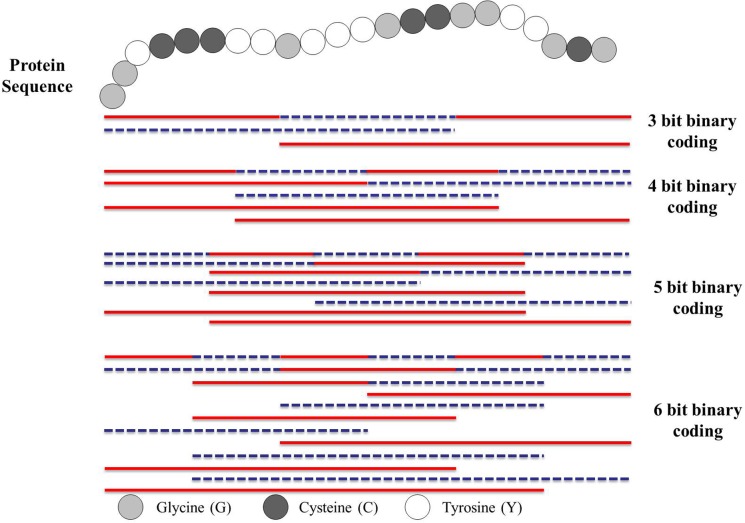
The Schematic diagram for constructing multi-scale local descriptor regions for a hypothetical protein sequence using 3–6 bit binary form. Each protein sequence is divided into multiple sub-sequences (segments) of varying length to represent multiple overlapping continuous segments.

In order to extract the interaction information, we first divide the entire protein sequence into a number of equal length segments. Then a novel binary coding scheme is adopted to construct a set of continuous regions on the basis of above partition. For example, consider a protein sequence “GGYCCCYYGYYYGCCGGYYGCG” containing 22 residues. To represent the sequence by a feature vector, let us first divide each protein sequence into multiple regions. Refer to Fig 9, the protein sequence is divided into four equal length segments (denoted by S_1_, S_2_, S_3_ and S_4_). Then it is encoded as a sequence of 1's and 0's of 4-bit binary form. In binary, these combinations are written as *0000*, *0001*, *0010*, *0011*, *0100*, *0101*, *0110*, *0111*, *1000*, *1001*, *1010*, *1011*, *1100*, *1101*, *1110*, *1111*. The number of states of a group of bits can be found by the expression 2^n^, where *n* is the number of bits. It should be noticed that here 0 or 1 denote one of the four equal length region S_1_—S_4_ is excluded or included in constructing the continuous regions respectively. For example, 0011 denotes a continuous region constructed by S_3_ and S_4_ (the final 50% of the sequence). Similarly, 0111 represents a continuous region constructed by S_2_, S_3_ and S_4_ (the final 75% of the sequence). These regions are illustrated in Fig 9.

It should be noticed that the proposed feature representation method can be simply and conveniently edited at multiple scales, which offers a promising new way for addressing aforementioned difficulties in a simple, unified, and theoretically sound way to represent protein sequence. For a given number of bits, each protein sequence may take on only a finite number of continuous regions. This limits the resolution of the sequence. If more bits are used for each protein sequence, then a higher degree of resolution is obtained. For example, if the protein sequence is encoded by 5-bit binary form, each protein sequence may take on 30 (2^5^–2) different regions. Higher bit encoding requires more storage for data and requires more computing resource to process. In this study, only the continuous regions are used and the discontinuous regions are discarded.

For each continuous region, three types of descriptors, composition (*C*), transition (*T*) and distribution (*D*), are used to represent its characteristics. *C* is the number of amino acids of a particular property (e.g., hydrophobicity) divided by the total number of amino acids in a local region. *T* characterizes the percentage frequency with which amino acids of a particular property is followed by amino acids of another property. *D* measures the chain length within which the first, 25%, 50%, 75%, and 100% of the amino acids of a particular property are located, respectively [50].

The three descriptors can be calculated in the following ways. Firstly, in order to reduce the complexity inherent in the representation of the 20 standard amino acids, we firstly clustered them into seven groups based on the dipoles and volumes of the side chains. Amino acids within the same groups likely involve synonymous mutations because of their similar characteristics [12].The amino acids belonging to each group are shown in Table 5.

**Table 5:** Division of amino acids into seven groups based on the dipoles and volumes of the side chains.

Group 1	Group 2	Group 3	Group 4	Group 5	Group 6	Group 7
A,G,V	C	M,S,T,Y	F,I,L,P	H,N,Q,W	K,R	D,E

Then, every amino acid in each protein sequence is replaced by the index depending on its grouping. For example, protein sequence “GGYCCCYYGYYYGCCGGYYGCG” is replaced by *1132223313331221133121* based on this classification of amino acids. There are eight ‘1’, six ‘2’ and eight ‘3’ in this protein sequence. The composition for these three symbols is 8/(8+6+8) ×100% = 36.36%, 6/(8+6+8) ×100% = 27.27% and 8/(8+6+8) ×100% = 36.36%, respectively. There are 4 transitions from ‘1’ to ‘2’ or from ‘2’ to ‘1’ in this sequence, and the percentage frequency of these transitions is (4/21) ×100% = 19%. The transitions from ‘1’ to ‘3’ or from ‘3’ to ‘1’ in this sequence can similarly be calculated as (6/21) ×100% = 28.57%. The transitions from ‘2’ to ‘3’ or from ‘3’ to ‘2’ in this sequence can also similarly be calculated as (2/21) ×100% = 9.52%.

**Fig 10:**
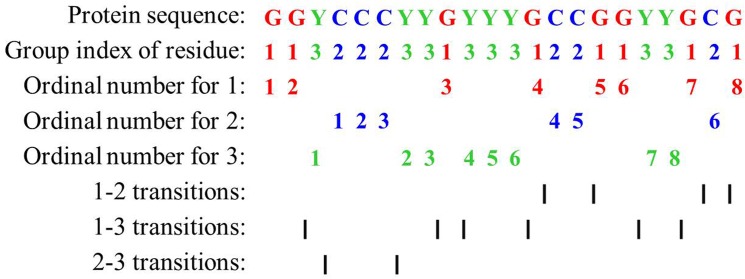
Sequence of a hypothetic protein indicating the construction of composition, transition and distribution descriptors of a protein region.

For distribution *D*, there are 8 residues encoded as “1” in the example of Fig 10, the positions for the first residue ‘1’, the 2nd residue ‘1’ (25% × 8 = 2), the *4th* ‘1’ residue (50% × 8 = 4), the *6th* ‘1’ (75% × 8 = 6) and the *8th* residue ‘1’ (100% × 8) in the encoded sequence are 1, 2, 13, 17, 22 respectively, so the *D* descriptors for ‘1’ are: (1/22) ×100% = 4.55%, (2/22) ×100% = 9.09%, (13/22) ×100% = 59.09%, (17/22) ×100% = 77.27%, (22/22)×100% = 100%, respectively. Similarly, the *D* descriptor for ‘2’ and ‘3’ is (18.18%, 18.18%, 27.27%, 63.64%, 95.45%) and (13.64%, 31.82%, 45.45%, 54.55%, 86.36%), respectively.

For each continuous local region, the three descriptors (*C*, *T* and *D*) are calculated and concatenated, and a total of 63 descriptors are generated: 7 for *C*, 21 ((7×6)/2) for *T* and 35 (7×5) for *D*. Then, all descriptors from 9 regions (4 bit) are concatenated and a total 567 dimensional vector has been built to represent each protein sequence. Finally, the PPI pair is characterized by concatenating the two vector spaces of two individual proteins. Thus, an 1134-dimentional vector has been constructed to represent each protein pair and used as a feature vector for input into RF classifier.

### Random Forest Classifier

Random Forest (RF) model is an ensemble classification algorithm that employs a collection of decision trees to reduce the output variance of individual trees and thus improves the stability and accuracy of classification. RF model is currently one of the most frequently employed machine learning techniques. RF takes advantage of two powerful machine-learning techniques: (1) the selection of training examples for each tree; (2) the random feature selection to split the data set. The first is performed by employing a bootstrap sample from original data (often referred to as bagging). Bagging works by sampling *n* samples with replacement from the original *n* samples, duplicating some examples and excluding some. The process results in two disjoint bags, one containing about 63.2% examples of the training data and one bag containing the rest which are usually denoted as out-of-bag (OOB) examples. In general, a random forest is constructed using the in bag examples and the OOB examples is used to estimate its prediction performance. The second feature selection procedure works by sampling a small subset of features at each node in each classification tree. More specifically, at each node of a tree, RF randomly selected a constant number of features and the one with the maximum decrease in Gini index is chosen for the split when growing the tree.

The RF model construction is composed of two parts, the ensemble creation and the tree generation. Specifically, the model construction requires a set of examples *S* = (((*x*_1_,*x*_2_,…,*x*_*n*_),*y*),…), where each example is described by a set of features *X* and a class label *y*; the number of trees to be constructed T_n_; and the number of features to examine at each split *F*_*n*_. In the ensemble creation step, *t* = 1,2,…,*T*_*n*_ trees are constructed from the in bag samples drawn with replacement from *S*. The tree construction algorithm starts by selecting *F*_*n*_ random features which can reduce the Gini index most if split upon. If no feature is found that reduce the error, a leaf is created predicting the most probable class from those examples reaching the node. Otherwise, the data is partitioned into two subsets: those for which the feature is positive and those for which it is negative. The partitions are subsequently used to recursively build new trees, with edges from the previous node. The recursion continues until there is no more informative feature, the node is pure or the total number of examples at the current node is minor 2. In the experiment we used the open source machine learning toolkit Weka to conduct this study.

## Supporting Information

S1 Dataset The names of protein pairs and their sequences of the PPIs dataset. In this dataset, the positive examples were downloaded from *S*.*cerevisiae* core subset of database of interacting proteins (DIP) and the negative examples were generated by selecting non-interacting pairs uniformly at random from the set of all proteins pairs that are not known to interact.
(XLSX)

S2 Dataset The names of protein pairs, their sequences and the subcellular localization information of the PPIs dataset. In this dataset, the positive examples were downloaded from *S*.*cerevisiae* core subset of database of interacting proteins (DIP) and the negative examples were generated by firstly selecting non-interacting pairs uniformly at random from the set of all proteins pairs that are not known to interact. Then the protein pairs with same subcellular localization information are excluded.
(XLSX)
